# When Literature Priors Are Removed: Clinical Rank Shifts and Transancestral Stability in Antipsychotic Low-Density Lipoprotein (LDL)-Risk Modelling

**DOI:** 10.7759/cureus.114952

**Published:** 2026-08-21

**Authors:** Ngo Cheung, Hoi-Ki Cheung, Yee-Wah Yu, Yolanda Yuen-Ching Tsang

**Affiliations:** 1 Psychiatry, Cheung Ngo Medical Limited, Hong Kong, HKG; 2 Psychiatry, Chinese University of Hong Kong, Hong Kong, HKG; 3 Psychiatry, University of Hong Kong, Hong Kong, HKG

**Keywords:** antipsychotic drugs, computational modelling, drug-ranking sensitivity, ki–ddd–twas, ldl cholesterol, metabolic risk, receptor pharmacology, receptor priors, transancestral stability, transcriptome-wide association studies

## Abstract

Antipsychotic-associated metabolic abnormalities vary considerably between drugs, but the receptor mechanisms contributing to lipid-related liability remain incompletely resolved. The integrated Ki-defined daily dose-transcriptome-wide association study (Ki-DDD-TWAS) score is a computational prioritization index derived from receptor-binding affinities, defined daily doses, and ancestry-stratified transcriptome-wide association study (TWAS) summary statistics; it is not a validated patient-level clinical risk predictor. We evaluated the sensitivity of an integrated Ki-DDD-TWAS prioritization framework to the removal of literature-derived receptor weights in ancestry-stratified low-density lipoprotein cholesterol (LDL) analyses. Saved outputs were available for 52 antipsychotics and five ancestry-defined LDL datasets: African, East Asian, European, Hispanic, and South Asian groups. Two models were compared: a literature-weighted model incorporating predefined metabolic receptor priors and a uniform model assigning a weight of 1.0 to all recognized receptor genes. Stage 3 analyses reconstructed receptor-level contributions and examined gene-direction consistency, ancestry-specific drivers, drug-rank correlations, rank stability, top-10 Jaccard similarity, leave-one-gene-out sensitivity, high-TWAS/low-weight candidates, and weighted-versus-uniform mean-rank differences.

Removing literature priors produced a large shift in relative reconstructed model contribution from histaminergic and serotonergic systems toward dopaminergic receptors. Dopaminergic contribution increased by 26.41 percentage points, whereas histaminergic contribution decreased by 16.76 percentage points. All 39 genes represented in the exported direction-consistency analysis changed in the same direction across all five ancestry datasets. Dopamine Receptor D2 (DRD2) was the leading gene-level contributor under the uniform model in every ancestry. Cross-ancestry drug-rank correlations were very high in both models, with mean off-diagonal Spearman correlations of 0.996 for the weighted model and 0.995 for the uniform model. The weighted model had complete top-10 set identity across ancestry pairs, whereas the mean top-10 Jaccard similarity under uniform weighting was 0.758. ADRB1 was the strongest high-TWAS/low-weight candidate, with a maximum absolute z-score of 6.079, but it met the candidate threshold in only one ancestry.

These findings indicate that literature-derived priors strongly shape model-based mechanistic attribution while exerting a smaller influence on broad drug ordering. The weighted model is best regarded as a clinically informed reference model, whereas the uniform model is a sensitivity and hypothesis-generation analysis. Because the five ancestry runs shared the same Ki matrix, defined daily dose (DDD) values, receptor map, and prior dictionary and lacked patient-level LDL validation, these findings describe computational model behavior rather than ancestry-specific clinical risk. Neither model is a validated clinical risk predictor, nor does either provide evidence of causal receptor mechanisms.

## Introduction

Clinical background of antipsychotic metabolic liability

Antipsychotic medications are essential treatments for schizophrenia, bipolar disorder, and other severe psychiatric conditions. Their use, however, is associated with a range of metabolic complications, including weight gain, obesity, glucose dysregulation, dyslipidemia, metabolic syndrome, and increased cardiovascular risk. These complications are clinically important because people with severe mental illness already experience higher rates of cardiometabolic disease and premature mortality than the general population. The metabolic consequences of treatment therefore occur in a population that may already have elevated baseline vulnerability.

The magnitude of metabolic liability is not uniform across antipsychotic drugs. Clinical trials, observational studies, systematic reviews, and network meta-analyses have consistently shown differences between individual agents. Clozapine and olanzapine are generally placed toward the less favorable end of the metabolic spectrum, with greater average adverse effects on weight, glucose, or selected lipid measures. In contrast, aripiprazole, ziprasidone, and lurasidone are usually associated with smaller average effects on weight or selected metabolic outcomes. These differences are not identical across all endpoints. A drug may have a relatively modest effect on weight but a different pattern of lipid or glucose changes, and estimates can vary according to treatment duration, patient population, baseline body mass index, comparator, and whether patients are antipsychotic-naive or switching from another agent [1-5].

The network meta-analysis [2] is particularly relevant to the present work because it included glucose, glycated hemoglobin, insulin resistance, triglycerides, total cholesterol, high-density lipoprotein (HDL) cholesterol, and LDL cholesterol. In that analysis, olanzapine and clozapine were associated with greater glyco-metabolic deterioration, whereas aripiprazole and ziprasidone were associated with smaller average deteriorations. The regression analysis also examined receptor occupancies and found that higher H1, M1, and M3 receptor occupancy was associated with larger glyco-metabolic changes. The earlier meta-analysis [1] similarly showed that the magnitude of antipsychotic-associated weight gain varies between agents and increases with longer exposure, although its analysis focused on weight and body mass index rather than LDL specifically.

The clinical importance of these effects has led to recommendations for baseline and follow-up monitoring of weight, glucose-related measures, and lipid abnormalities in patients receiving antipsychotic treatment. The consensus statement from the American Diabetes Association and collaborating organizations emphasized the need to recognize obesity and diabetes risk during antipsychotic treatment [6], while later clinical guidance addressed the management of weight gain, metabolic disturbances, and cardiovascular risk [7,8]. These recommendations provide the clinical context for computational prioritization, but they do not establish that a receptor-based score can substitute for patient-level monitoring or outcome-based risk prediction.

Receptor mechanisms

Receptor pharmacology has long been used to explain why antipsychotic drugs differ in their adverse-effect profiles. Histamine H1 receptor affinity is one of the best-known pharmacological correlates of antipsychotic-associated weight gain. A receptor-binding analysis reported that H1-histamine receptor affinity predicted short-term weight gain across typical and atypical antipsychotics [9]. The analysis also identified associations involving α1A-adrenergic, 5-HT2C, and 5-HT6 receptor affinities. These findings helped establish the rationale for assigning greater prior importance to HRH1 and HTR2C in metabolic-risk models.

The underlying biology is nevertheless multireceptor and context-dependent. Antipsychotics often bind to several receptor systems simultaneously, and their downstream effects depend on receptor occupancy, tissue distribution, intrinsic activity, dose, treatment duration, and individual susceptibility. Serotonergic, histaminergic, muscarinic, adrenergic, and dopaminergic pathways can influence appetite, energy balance, insulin secretion, glucose transport, lipid metabolism, and central reward processing. Reviews of antipsychotic metabolic effects have therefore emphasized that no single receptor fully explains the clinical phenotype [10-12]. The multiplicity of serotonin receptors provides an additional reason to treat receptor-level attribution as context-dependent rather than as a direct statement of causal importance [13].

The contribution of a receptor in a computational model should also be distinguished from its causal importance in patients. A receptor may receive a high prior weight but contribute little to a particular drug score if the available binding measurements, dose proxy, or transcriptome-wide association study (TWAS) signal are weak. Conversely, a receptor with a low or absent prior weight may contribute substantially under a uniform model because of its affinity profile or the transformation applied to the genetic association signal. Quantifying this distinction is the central purpose of the present analysis.

Genomic context and multi-ancestry considerations

LDL cholesterol is a clinically relevant lipid trait with a substantial genetic component. Multi-ancestry lipid studies have demonstrated both shared and ancestry-dependent features of lipid-associated genetic architecture. Differences in allele frequencies, linkage disequilibrium, effect sizes, imputation quality, and fine-mapping resolution can affect the apparent strength and location of genetic associations. The Global Lipids Genetics Consortium has shown that genetic diversity can improve the discovery and localization of lipid-associated loci, while also highlighting the importance of including populations that have historically been underrepresented in genome-wide association studies [14].

Transcriptome-wide association studies provide a complementary way to connect genetic variation with gene expression and complex traits. By estimating genetically regulated expression and testing its association with a phenotype, TWAS methods can prioritize genes and tissues that may lie on relevant biological pathways. However, a TWAS association does not automatically establish that the implicated gene is causal. The association may arise because the expression-prediction model is correlated with a causal variant acting through another mechanism. After all, several genes have correlated prediction models, or because of linkage disequilibrium between the predicted-expression signal and an independent causal locus. Tissue mismatch, prediction uncertainty, ancestry mismatch, and differences in linkage-disequilibrium structure also complicate interpretation [15-18].

These considerations are especially important when ancestry-specific TWAS results are integrated into a pharmacological score. Similar rankings across ancestry datasets may indicate shared biological signal, but they may also reflect the common pharmacological component of the model. Conversely, ancestry-specific candidate genes may reflect genuine population-specific genetic architecture, differences in prediction accuracy, or stochastic variation in the underlying summary statistics. The present study therefore treats ancestry comparisons as an analysis of model stability and sensitivity rather than as a direct test of ancestry-specific clinical risk.

Prior work in the present series

The present analysis forms part of a broader series examining the behavior of an integrated Ki-defined daily dose-transcriptome-wide association study (Ki-DDD-TWAS) framework. The first study compared four transformations for incorporating TWAS evidence into antipsychotic metabolic-risk scores. It found that changing the TWAS transformation primarily altered score magnitude while preserving much of the drug ordering [19]. The second study removed literature-derived receptor weights and showed that the broad ranking could remain relatively stable even when the receptor-level explanation changed substantially. That analysis also identified high-TWAS/low-weight candidates, including ADRB1, as hypothesis-generating signals [20]. The third study examined receptor-prior sensitivity in HDL-related analyses across multiple ancestry datasets and described both transancestral stability and clinical discordance [21].

In this paper, “literature-derived receptor priors” means predefined numerical weights assigned to selected metabolic receptors on the basis of prior pharmacological literature. “Ki-DDD-TWAS” denotes a composite score combining inverse Ki, a log-transformed defined-daily-dose term, a receptor weight, and a TWAS-derived scaling factor.

The present paper focuses on LDL and extends the Stage 3 analysis to a second major lipid trait. The central question is whether the prior-sensitivity pattern observed in the earlier work is reproducible when ancestry-stratified LDL TWAS outputs are substituted into the same pharmacological framework. The LDL-specific prior-sensitivity comparison and Stage 3 synthesis are the analyses added here; the LDL TWAS files and previously generated receptor-level and drug-level outputs were reused. The analysis is deliberately downstream. It uses saved long-format and drug-level outputs and does not introduce a new genome-wide association study (GWAS), TWAS, receptor-binding, or causal-inference method.

Aim

The primary objective was to quantify the sensitivity of an integrated Ki-DDD-TWAS prioritization framework to the removal of literature-derived receptor priors in ancestry-stratified LDL analyses. The secondary aims were to quantify changes in receptor-class and gene-level contribution when literature-derived metabolic weights were removed; evaluate the transancestral robustness of drug rankings and gene-direction changes across five ancestry-defined LDL datasets; place the weighted ranking in clinical context and characterize rank movements produced by uniform weighting without treating those movements as clinical validation; identify high-TWAS/low-weight candidates and determine whether they were supported across more than one ancestry; and assess whether the evidence justified dual reporting of a literature-weighted reference model and a uniform sensitivity model.

## Materials and methods

Data sources

**Figure 1:**
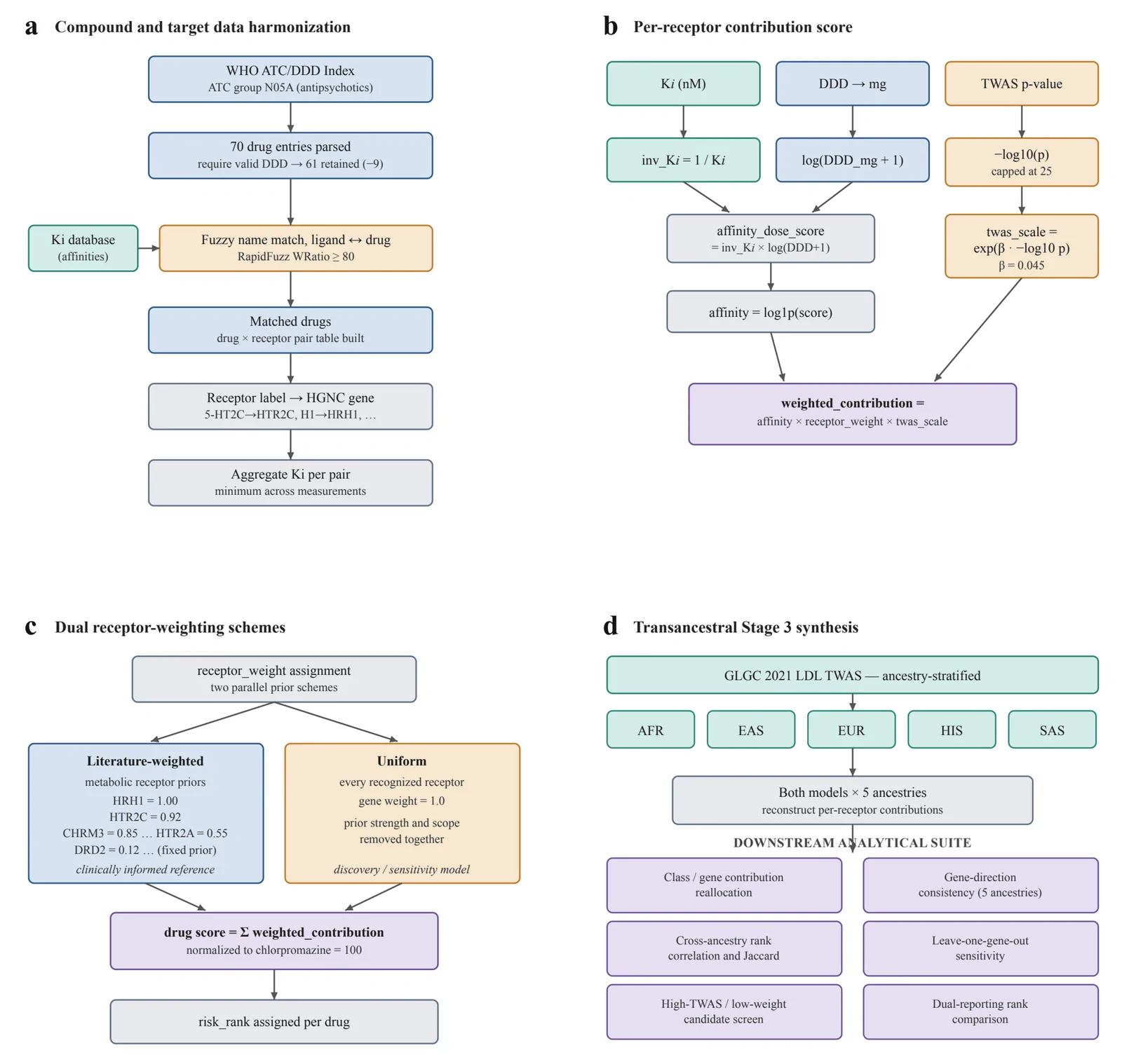
Experimental pipeline for prior-sensitivity analysis of the antipsychotic Ki–DDD–TWAS framework (a) Compound and target data harmonization. Antipsychotic entries are parsed from the WHO ATC/DDD Index (group N05A) [21], filtered to those with a valid defined daily dose (DDD), and matched to curated Ki-database ligands by fuzzy name matching (WRatio ≥ 80) [22]. Matched pairs form the drug × receptor table; receptor labels are mapped to HGNC gene symbols and Ki values are aggregated per pair by the minimum across measurements. (b) Per-receptor contribution score. Inverse Ki and the log-transformed DDD are combined into the affinity–dose score, log1p-transformed to give the affinity term. The TWAS p-value is converted to a capped −log10(p) and an exponential scaling factor (β = 0.045); the weighted contribution is the product of affinity, receptor weight and TWAS scale. (c) Dual receptor-weighting schemes. Contributions are computed under a literature-weighted model (fixed metabolic receptor priors, clinically informed reference) and a uniform model (all recognized receptor genes = 1.0, discovery/sensitivity), then summed per drug, normalized to chlorpromazine = 100, and ranked. (d) Transancestral Stage 3 synthesis. Both models are run across five ancestry-stratified GLGC 2021 LDL TWAS datasets [13]; per-receptor contributions are reconstructed from saved outputs and passed through the downstream analytical suite. This figure illustrates the workflow only and does not depict results. Ki: inhibition constant; DDD: defined daily dose; TWAS: transcriptome-wide association study; WHO: World Health Organization; ATC: Anatomical Therapeutic Chemical; HGNC: HUGO Gene Nomenclature Committee; WRatio: weighted ratio; GLGC: Global Lipids Genetics Consortium; LDL: low-density lipoprotein cholesterol; p: p-value; β: beta scaling coefficient; −log10(p): negative base-10 logarithm of the p-value.

This study used saved outputs from an integrated antipsychotic pharmacology and lipid-TWAS pipeline (Figure 1). The present Stage 3 analysis reused previously generated long-format receptor-level and drug-level outputs; it did not generate new GWAS, TWAS, receptor-binding, or patient-level clinical data. The antipsychotic drug list was generated from the N05A parent group of the World Health Organization Anatomical Therapeutic Chemical Classification System and Defined Daily Dose Index [22]. The initial list contained 70 drug entries. Nine drugs were subsequently excluded because they lacked a valid defined daily dose, leaving 61 entries for further processing. Fifty-eight drugs were matched to ligand names in the Ki database using fuzzy name matching with a threshold of 80.

The Ki database was an internally curated ligand-receptor dataset identified as KiDatabase_2026-06-22.csv [23]. Ki values were parsed as nanomolar measurements. When multiple measurements were available for the same drug-receptor pair, the minimum observed Ki value was used, corresponding to the strongest recorded binding measurement. Minimum-Ki aggregation was retained because the prespecified pipeline was intended to capture the strongest recorded interaction; a median or geometric-mean Ki sensitivity analysis was not included in the saved Stage 3 run. Defined daily doses were converted to milligrams. The affinity-dose term was then calculated from inverse Ki and the natural logarithm of the milligram dose plus one.

The genetic component consisted of precomputed ancestry-stratified LDL TWAS outputs derived from the GLGC 2021 resource [14]. Five ancestry-defined low-density lipoprotein cholesterol datasets were analyzed: African ancestry (LDL_AFR), East Asian ancestry (LDL_EAS), European ancestry (LDL_EUR), Hispanic ancestry (LDL_HIS), and South Asian ancestry (LDL_SAS). The labels are retained from the supplied data files. The ancestry-specific files contained between 17,620 and 18,057 genes. Each completed pipeline run contained 1,285 drug-receptor pairs. The downstream analysis used only the saved long-format receptor-level files and drug-level risk-score files. No new drug matching, Ki lookup, or TWAS calculation was performed during Stage 3.

Score construction

The upstream affinity-dose score was defined as inverse Ki multiplied by the natural logarithm of defined daily dose in milligrams plus one. Before receptor weighting, this quantity was transformed using the natural logarithm of one plus the affinity-dose score. For each receptor-gene row, the transformed affinity component was multiplied by a receptor weight and by a TWAS scaling factor.

The TWAS scaling factor was calculated as the exponential of 0.045 multiplied by the negative log10 of the TWAS p-value. β = 0.045 was retained from the earlier TWAS-transformation study [19] to provide mild, rather than dominant, amplification. Under this formula, a p-value of 10−5 gives a multiplier of approximately 1.25, while a negative-log10 p-value of 25 gives a maximum multiplier of approximately 3.08. β was not estimated or clinically calibrated in the present LDL analysis. Negative log10 p-values were capped at 25 to prevent extreme amplification. The saved Stage 3 outputs did not include a separate cap-sensitivity sweep, so sensitivity to this cap was not formally estimated here. The saved pipeline also retained a secondary drug-level multiplier based on the maximum absolute TWAS z-score and a nominal-significance multiplier for drug-receptor rows with p-values below 0.05. The nominal p-value threshold was exploratory and was not adjusted for the number of genes, drugs, receptor rows, ancestries, or traits.

Drug-level scores were normalized to chlorpromazine, which was set to 100. This normalization is a reference convention and does not represent a clinical relative risk, incidence rate, probability, or percentage change in LDL. Because the per-receptor transformation used p-values and the secondary multiplier used absolute z-scores, the score emphasized statistical strength and magnitude but did not preserve the direction of the TWAS association. A strong positive and a strong negative LDL association could therefore increase a score in a similar way. The resulting score is therefore direction-agnostic and should not be interpreted as a directional estimate of LDL liability or a causal mediation score.

Two versions of the model were analyzed. The literature-weighted model used predefined metabolic receptor weights. The largest weights were assigned to HRH1, HTR2C, and CHRM3, with values of 1.00, 0.92, and 0.85, respectively. HTR2A received a weight of 0.55. DRD3, DRD2, and DRD4 received weights of 0.18, 0.12, and 0.10. Other receptors were assigned lower weights according to the supplied dictionary.

The uniform model assigned a weight of 1.0 to every recognized receptor gene present in the scored long-format data. It therefore did more than remove differential weighting among the genes already represented in the literature-weight dictionary. It also restored contribution from recognized receptor genes that had no positive literature weight in the weighted model, including genes such as HTR2B and DRD1. The uniform model should consequently be regarded as an equal-weight prior model, not as an absence of pharmacological assumptions.

Stage 3 analytical suite

The Stage 3 analysis reconstructed per-receptor contributions using the saved affinity-dose, TWAS p-value, and gene-mapping fields. Missing p-values were handled using the prespecified fairness-imputation rule, in which missing values among scored genes were replaced with the mean negative log10 p-value available in the relevant run. This rule prevented missingness from automatically setting a receptor contribution to zero, but it also assigned a common value to rows without directly observed p-values. The upstream outputs recorded 210 TWAS-imputed rows in the African, European, Hispanic, and South Asian datasets and 239 in the East Asian dataset. A separate exclusion-based sensitivity analysis was not included in the saved Stage 3 run. This reconstruction was intended to reproduce the contribution formula and did not involve re-matching drugs or re-querying the Ki database.

The analysis first pooled receptor contributions across the five ancestry datasets and calculated the percentage of total contribution attributable to each receptor class. Gene-level contribution percentages were then calculated separately for each ancestry and compared between the weighted and uniform models. The difference was defined as the uniform contribution share minus the weighted contribution share. Directional consistency was defined as the fraction of ancestry datasets showing the same sign of change.

Additional analyses included ancestry-specific rankings of the leading uniform-model genes, Spearman correlations of drug ranks between ancestry datasets, the mean rank and standard deviation of each drug across ancestries, and pairwise Jaccard similarity of the top-10 drug sets. Leave-one-gene-out analyses were performed in the weighted model for HRH1, HTR2C, CHRM3, HTR2A, DRD2, ADRA1A, HTR6, and HTR7. These analyses removed the selected gene’s reconstructed contribution and recalculated the rank from the remaining contribution. They did not fully recompute every secondary drug-level multiplier used in the saved final score.

The high-TWAS/low-weight candidate screen used a strict threshold of an absolute z-score of at least 3.0 and literature weight no greater than 0.15 in the downstream analysis. The Stage 3 synthesis also used a more permissive exploratory threshold of an absolute z-score at least 2.5 with the same weight ceiling. Candidate support was counted by the number of ancestry datasets in which a gene met the threshold. All candidate-gene findings were treated as exploratory model signals rather than as evidence of causal or clinical relevance. A dual-reporting analysis compared each drug’s mean rank under the weighted and uniform models.

Software and reproducibility

All Stage 3 analyses were performed using Python (Python Software Foundation, Wilmington, Delaware, USA) with the open-source packages pandas, NumPy, RapidFuzz, tqdm, and SciPy. The openpyxl package was used to export the spreadsheet. The analyses used saved output files and reconstructed contributions without repeating drug-name matching or receptor lookup. The output directories contained the long-format receptor-level files, drug-level risk files, and supporting Stage 3 summaries. 

## Results

Data coverage and pipeline output characteristics

All five weighted and five uniform ancestry runs contained both long-format receptor-level data and drug-level risk files. The ancestry-specific TWAS directories contained between 17,620 and 18,057 genes. Each run included 70 drugs in the initial list, nine drugs dropped for missing defined daily dose, 58 matched ligands, and 1,285 drug-receptor pairs (Table 1).

**Table 1:** Cross-run LDL pipeline coverage and output characteristics Values are pipeline counts and computational outputs, not clinical risk estimates. DDD: defined daily dose, Ki: inhibition constant; TWAS: transcriptome-wide association study; LDL_AFR: low-density lipoprotein cholesterol, African ancestry; LDL_EAS: low-density lipoprotein cholesterol, East Asian ancestry; LDL_EUR: low-density lipoprotein cholesterol, European ancestry; LDL_HIS: low-density lipoprotein cholesterol, Hispanic ancestry; LDL_SAS: low-density lipoprotein cholesterol, South Asian ancestry.

Mode	Ancestry	Genes with TWAS data	Drugs in input list	Drugs dropped for no DDD	Drugs matched to ligand	Drug × receptor pairs	Ki values imputed	TWAS values imputed	Strong binders	TWAS-significant rows	Receptor genes without TWAS
Weighted	LDL_AFR	18,016	70	9	58	1,285	0	210	247	111	9
Weighted	LDL_EAS	17,620	70	9	58	1,285	0	239	247	97	10
Weighted	LDL_EUR	18,057	70	9	58	1,285	0	210	247	150	9
Weighted	LDL_HIS	18,017	70	9	58	1,285	0	210	247	147	9
Weighted	LDL_SAS	17,936	70	9	58	1,285	0	210	247	175	9
Uniform	LDL_AFR	18,016	70	9	58	1,285	0	210	247	111	9
Uniform	LDL_EAS	17,620	70	9	58	1,285	0	239	247	97	10
Uniform	LDL_EUR	18,057	70	9	58	1,285	0	210	247	150	9
Uniform	LDL_HIS	18,017	70	9	58	1,285	0	210	247	147	9
Uniform	LDL_SAS	17,936	70	9	58	1,285	0	210	247	175	9

No rows were removed because the affinity-dose score was zero, and no Ki values were imputed in the reported runs. TWAS values were imputed for 210 rows in the African, European, Hispanic, and South Asian datasets and for 239 rows in the East Asian dataset. The number of nominally significant drug-receptor rows varied from 97 in the East Asian dataset to 175 in the South Asian dataset. Between nine and ten receptor genes lacked direct TWAS data in each ancestry run (Table 1).

These figures indicate that the planned computational outputs were available across all ancestry and weighting conditions. They are workflow-coverage metrics and should not be interpreted as evidence of clinical representativeness or biological completeness. In particular, missing TWAS values, deterministic receptor-to-gene mapping, and the restricted receptor set remain relevant to the interpretation of contribution shares.

Receptor-class and gene-level reallocation

The clearest difference between the two models was a redistribution of relative reconstructed model contribution across receptor classes. In the literature-weighted model, the pooled reconstructed contribution share of serotonergic receptors was 40.75%, histaminergic receptors 25.46%, dopaminergic receptors 17.61%, adrenergic receptors 12.91%, muscarinic receptors 3.14%, transporters 0.07%, and sigma receptors 0.06%. Under uniform weighting, the reconstructed dopaminergic share increased to 44.02%, representing a gain of 26.41 percentage points. Histaminergic contribution fell to 8.70%, a loss of 16.76 percentage points. Serotonergic contribution decreased to 32.70%, a loss of 8.05 percentage points. Adrenergic contribution decreased by 2.62 percentage points to 10.29%. Muscarinic, transporter, and sigma contributions changed only modestly (Table 2).

**Table 2:** Receptor-class and representative gene-level contribution reallocation after removal of literature priors Class-level values are pooled contribution shares. Gene-level values are mean uniform-minus-weighted contribution differences across the five ancestry datasets. Consistency is the fraction of ancestry datasets showing the same direction of change. Values are computational contribution measures, not clinical mediation estimates.

Level	Receptor/gene	Class	Weighted contribution (%)	Uniform contribution (%)	Delta or mean delta (percentage points)	Consistency
Class	Dopaminergic	Dopaminergic	17.61	44.02	+26.41	—
Class	Histaminergic	Histaminergic	25.46	8.70	−16.76	—
Class	Serotonergic	Serotonergic	40.75	32.70	−8.05	—
Class	Adrenergic	Adrenergic	12.91	10.29	−2.62	—
Class	Muscarinic	Muscarinic	3.14	3.46	+0.32	—
Class	Transporter	Transporter	0.07	0.44	+0.37	—
Class	Sigma	Sigma	0.06	0.40	+0.34	—
Gene	DRD2	Dopaminergic	—	—	+13.326	1.00
Gene	DRD4	Dopaminergic	—	—	+5.894	1.00
Gene	DRD3	Dopaminergic	—	—	+5.838	1.00
Gene	HTR2B	Serotonergic	—	—	+3.754	1.00
Gene	HTR1A	Serotonergic	—	—	+1.598	1.00
Gene	HTR7	Serotonergic	—	—	+1.124	1.00
Gene	DRD1	Dopaminergic	—	—	+1.026	1.00
Gene	ADRA1A	Adrenergic	—	—	−2.048	1.00
Gene	HRH1	Histaminergic	—	—	−17.175	1.00
Gene	HTR2A	Serotonergic	—	—	−9.265	1.00
Gene	HTR2C	Serotonergic	—	—	−6.599	1.00
Gene	HTR6	Serotonergic	—	—	−0.802	1.00
Gene	CHRM3	Muscarinic	—	—	−0.724	1.00

The gene-level analysis showed that this was not only a class-level effect. At the model level, DRD2 gained 13.326 percentage points of relative contribution, while DRD4 and DRD3 gained 5.894 and 5.838 points, respectively. Other consistently gaining genes included HTR2B, HTR1A, HTR7, DRD1, HTR1D, HTR1B, HTR3A, CHRM2, ADRA1D, HRH2, SIGMAR1, and SLC6A2. The largest losses were observed for HRH1, which declined by 17.175 points, HTR2A, which declined by 9.265 points, and HTR2C, which declined by 6.599 points. ADRA1A, ADRA1B, HTR6, and CHRM3 also lost relative contribution (Table 2).

These percentages describe the allocation of the model’s reconstructed signal. They should not be interpreted as estimates of the percentage of clinical LDL risk mediated by a receptor or receptor class. The reallocation is mathematically expected to some extent because the weighted model gives much greater initial importance to HRH1 and HTR2C than to DRD2, DRD3, or DRD4. It is nevertheless informative because it identifies which model-based biological explanations become prominent when those priors are removed.

Transancestral directional consistency and dominant model contributors

All 39 genes represented in the exported gene-direction consistency file showed the same direction of model contribution change across the five ancestry-defined LDL datasets. DRD2, DRD3, and DRD4 gained relative contribution in every ancestry. HRH1, HTR2A, and HTR2C lost relative contribution in every ancestry. The same directional consistency was observed for the smaller changes affecting adrenergic, muscarinic, serotonergic, histaminergic, sigma, and transporter genes (Table 2).

Under uniform weighting, DRD2 was the leading gene by reconstructed contribution in all five ancestry datasets. DRD3 occupied second place in the African and European datasets, whereas HTR2A occupied second place in the East Asian, Hispanic, and South Asian datasets. HTR2A and DRD3 exchanged positions near the top of the ranking, but DRD2 remained the number-one contributor in every dataset. DRD4, HRH1, HTR7, ADRA1A, and HTR2B or HTR2C also appeared among the leading model contributors.

The perfect directional consistency and universal DRD2 leadership indicate that the broad reallocation pattern was not driven by one ancestry dataset. However, the five analyses shared the same Ki matrix, defined daily dose values, receptor-to-gene map, and prior-weight comparison. The consistency therefore reflects the combined influence of ancestry-specific TWAS information and a common pharmacological structure. It demonstrates reproducibility of model sensitivity rather than independent confirmation of causal receptor biology. These findings also do not constitute evidence of ancestry-specific clinical LDL risk.

Transancestral stability of drug rankings

Drug ordering was highly correlated across ancestry datasets in both weighting modes. The weighted model had a mean off-diagonal Spearman correlation of 0.996. Pairwise correlations ranged from 0.991 to 0.998. The uniform model had a mean off-diagonal Spearman correlation of 0.995, with pairwise values ranging from 0.993 to 0.997 (Table 3).

**Table 3:** Summary of cross-ancestry stability

Mode	Mean off-diagonal Spearman rho	Pairwise rho range	Mean pairwise top-10 Jaccard	Jaccard range
Weighted	0.996	0.991–0.998	1.000	1.000–1.000
Uniform	0.995	0.993–0.997	0.758	0.667–0.818

The weighted model also showed complete top-10 set identity across every ancestry pair. Every pairwise weighted top-10 Jaccard value was 1.000. The uniform model retained substantial overlap but was less stable, with a mean pairwise top-10 Jaccard value of 0.758. Individual uniform-model Jaccard values ranged from 0.667 to 0.818. The reduction in top-10 overlap indicates that the literature priors stabilized membership of the highest-ranked set, even though the overall rank correlations remained very high without them.

The weighted model produced perfect rank stability across the five datasets for several drugs, including clozapine, olanzapine, aripiprazole, brexpiprazole, iloperidone, cariprazine, chlorpromazine, melperone, levosulpride, triflupromazine, sultopride, and thioridazine. Sulpiride and amisulpride were among the least stable weighted-model drugs, each with a rank standard deviation of 2.17. Under uniform weighting, clozapine, aripiprazole, fluphenazine, perphenazine, levosulpiride, zotepine, and thioridazine had standard deviations of zero. Sulpiride had the largest uniform-model standard deviation, at 2.74, followed by chlorprothixene at 2.51 (Table 4).

**Table 4:** Selected drug-level rank stability Rank 1 denotes the highest model-predicted risk. Jaccard values refer to the pairwise overlap of the top-10 drug sets. SD: standard deviation.

Drug	Weighted mean rank	Weighted rank SD	Uniform mean rank	Uniform rank SD
Clozapine	1.00	0.00	1.00	0.00
Aripiprazole	20.00	0.00	14.00	0.00
Fluphenazine	13.80	0.84	15.00	0.00
Perphenazine	13.80	1.30	16.00	0.00
Zotepine	4.40	1.14	6.00	0.00
Thioridazine	8.00	0.00	7.00	0.00
Ziprasidone	5.60	0.55	2.40	0.55
Olanzapine	7.00	0.00	12.00	1.41
Quetiapine	14.80	0.84	25.20	1.48
Sulpiride	31.20	2.17	22.00	2.74
Chlorprothixene	9.20	0.45	10.40	2.51

The delta-rank analysis provided a related measure of transancestral consistency. Across 52 common drugs, pairwise Spearman correlations of the uniform-minus-weighted rank-change profiles ranged from 0.931 to 0.976. All pairwise comparisons were associated with very small p-values. Thus, ancestry-specific LDL inputs produced not only similar absolute rankings but also similar responses to removal of the literature priors.

Clinical context and rank movements under prior removal

The weighted rankings were qualitatively concordant with selected clinical patterns at the highest-liability end, but this was not an external clinical validation. Clozapine was ranked first in all five ancestry datasets, with a mean rank of 1.00 and a rank standard deviation of zero. Olanzapine had a mean weighted rank of 7.00 and also showed no ancestry-related rank variation. Aripiprazole had a mean rank of 20.00 and a standard deviation of zero. Ziprasidone had a mean weighted rank of 5.60 and a standard deviation of 0.55. The model therefore preserved the prominence of clozapine and placed olanzapine in the upper tier, while aripiprazole was lower in the predicted-risk ordering. Ziprasidone was more intermediate than its generally favorable clinical and metabolic profile might suggest.

Because rank 1 denotes the highest predicted risk, the sign of the rank change requires careful interpretation. A negative value for uniform rank minus weighted rank means that the drug moved toward a numerically smaller rank and therefore toward a higher predicted-risk position under uniform weighting. A positive value means that the drug moved toward a numerically larger rank and therefore toward a lower predicted-risk position.

Several drugs with relatively favorable clinical metabolic profiles moved toward numerically smaller ranks under uniform weighting. Ziprasidone changed from a mean weighted rank of 5.60 to a mean uniform rank of 2.40. Aripiprazole changed from 20.00 to 14.00, and haloperidol changed from 17.80 to 9.60. Pimozide, cariprazine, amisulpride, and several dopamine-focused older agents also moved toward higher predicted-risk positions under the uniform model. These movements should not be described as clinical improvement; they represent increased model-predicted risk after the priors were removed (Table 5).

**Table 5:** ​​​​​​​Largest mean rank movements

Movement group	Drug	Mean delta rank, uniform − weighted
Biggest risers	Levomepromazine	+20.00
Biggest risers	Acepromazine	+16.80
Biggest risers	Promazine	+16.80
Biggest risers	Loxapine	+11.80
Biggest risers	Quetiapine	+10.40
Biggest risers	Periciazine	+8.40
Biggest risers	Clotiapine	+7.40
Biggest risers	Paliperidone	+7.00
Biggest risers	Pipamperone	+6.40
Biggest risers	Mesoridazine	+6.00
Biggest fallers	Amisulpride	−9.40
Biggest fallers	Cariprazine	−9.40
Biggest fallers	Thioproperazine	−9.20
Biggest fallers	Trifluperidol	−9.20
Biggest fallers	Sulpiride	−9.20
Biggest fallers	Benperidol	−9.00
Biggest fallers	Haloperidol	−8.20
Biggest fallers	Moperone	−8.00
Biggest fallers	Pipotiazine	−8.00
Biggest fallers	Sultopride	−6.80

Other drugs moved toward numerically larger ranks under uniform weighting. Olanzapine changed from a mean weighted rank of 7.00 to 12.00, quetiapine from 14.80 to 25.20, levomepromazine from 18.00 to 38.00, loxapine from 18.20 to 30.00, promazine from 23.00 to 39.80, and acepromazine from 25.40 to 42.20. The largest positive mean shifts were observed for levomepromazine, acepromazine, promazine, loxapine, quetiapine, periciazine, clotiapine, paliperidone, pipamperone, and mesoridazine. The largest negative shifts were observed for amisulpride, cariprazine, thioproperazine, trifluperidol, sulpiride, benperidol, haloperidol, moperone, pipotiazine, and sultopride (Table 5).

These movements suggest that the weighted model’s greater emphasis on HRH1 and HTR2C helps preserve the relative prominence of drugs with greater reported average adverse effects on weight, glucose, or selected lipid measures, although the model is not a full clinical ranking. Conversely, uniform weighting makes dopaminergic binding more influential and produces several movements that are discordant with the commonly described clinical metabolic profiles of aripiprazole, ziprasidone, and haloperidol. This discordance is informative as a sensitivity result but does not establish that the uniform model is biologically more accurate.

Leave-one-gene-out sensitivity

Removing individual genes from the reconstructed weighted contributions identified HRH1 as the most ranking-sensitive component in the reconstructed weighted model. The mean absolute rank shift across drugs and ancestry datasets was 4.58 positions for HRH1. The corresponding values were 3.32 for HTR2A, 2.14 for DRD2, 1.76 for HTR7, 1.55 for HTR2C, 1.44 for ADRA1A, 1.31 for HTR6, and 1.17 for CHRM3 (Table 6).

**Table 6:** Leave-one-gene-out sensitivity

Gene	Mean absolute rank change
HRH1	4.58
HTR2A	3.32
DRD2	2.14
HTR7	1.76
HTR2C	1.55
ADRA1A	1.44
HTR6	1.31
CHRM3	1.17

In the European dataset, removing HRH1 moved clotiapine and paliperidone eight positions toward numerically smaller or higher predicted-risk ranks. Moperone and lurasidone also shifted by eight positions. Periciazine, benperidol, trifluoperazine, and pipamperone shifted by seven positions, while pimozide and pipotiazine shifted by six and five positions, respectively.

These results identify dependence of the reconstructed ranking on particular receptor components. HRH1 was the strongest ranking-sensitive component in this analysis, which is consistent with the high weight assigned to HRH1 and with the established pharmacological association between H1 receptor affinity and antipsychotic-associated weight gain [9]. The leave-one-gene-out analysis should nevertheless be interpreted as a model-dependence analysis, not as evidence that HRH1 independently causes LDL abnormalities. In addition, the reported reconstruction removed receptor-level contributions but did not fully recompute every drug-level multiplier used in the saved final score. The results are therefore best described as sensitivity of the reconstructed contribution-based ranking.

High-TWAS/low-weight candidates and dual reporting

Under the strict downstream screen of an absolute z-score of at least 3.0 and literature weight no greater than 0.15, ADRB1 and SLC6A3 were identified. These were exploratory, single-ancestry signals rather than replicated candidate genes. ADRB1 had a maximum absolute z-score of 6.079, a literature weight of 0.08, and appeared in 20 drug-level records. SLC6A3 had a maximum absolute z-score of 3.384, a literature weight of 0.04, and appeared in 29 drug-level records (Table 7).

**Table 7:** High-TWAS/low-weight candidates

Gene	Screen	Maximum absolute TWAS z	Literature weight	Number of drugs	Number of ancestries
ADRB1	Strict and exploratory	6.079	0.08	20	1
SLC6A3	Strict and exploratory	3.384	0.04	29	1
SLC6A4	Exploratory	2.902	0.06	9	1
DRD4	Exploratory	2.698	0.10	39	1
HTR2B	Exploratory	2.696	0.00	17	1

The more permissive Stage 3 threshold also identified SLC6A4, DRD4, and HTR2B. Each candidate was supported in only one ancestry dataset. HTR2B had no positive value in the supplied literature-weight dictionary, so its uniform-model contribution reflects restoration of a gene that was not active in the weighted model. The absence of a candidate supported across multiple ancestry datasets contrasts with the strong transancestral consistency of the broad receptor-reallocation pattern.

The dual-reporting analysis showed that clozapine remained ranked first under both models. Other drugs displayed more substantial changes. Ziprasidone moved from a weighted mean rank of 5.60 to 2.40 under uniform weighting, whereas olanzapine moved from 7.00 to 12.00 and quetiapine from 14.80 to 25.20. The dual-reporting result therefore separates two uses of the framework. The weighted model offers the more clinically interpretable reference ordering because it preserves established receptor knowledge, although this represents qualitative concordance rather than external validation. The uniform model reveals how the ranking and mechanistic explanation change when those priors are removed (Table 8).

**Table 8:** Selected dual-reporting rank shifts TWAS, transcriptome-wide association study. Rank 1 denotes the highest model-predicted risk. A positive rank shift indicates movement toward a numerically larger rank; a negative rank shift indicates movement toward a numerically smaller rank. Values are computational outputs, not clinical risk estimates.

Drug	Weighted mean rank	Uniform mean rank	Rank shift, uniform − weighted
Clozapine	1.00	1.00	0.00
Ziprasidone	5.60	2.40	−3.20
Olanzapine	7.00	12.00	+5.00
Quetiapine	14.80	25.20	+10.40
Aripiprazole	20.00	14.00	−6.00
Haloperidol	17.80	9.60	−8.20
Levomepromazine	18.00	38.00	+20.00

Additional detailed outputs, including full ancestry-specific drug ranks, complete receptor- and gene-level contribution tables, pairwise rank-correlation and Jaccard matrices, leave-one-gene-out results, and the full high-TWAS/low-weight candidate listings, are provided in Appendix 1. 

## Discussion

Transancestral robustness

The five ancestry runs shared the same Ki matrix, DDD values, receptor-to-gene map, drug list, and literature-derived prior dictionary. Accordingly, consistency across runs demonstrates structural reproducibility of model behavior, not independent biological replication or ancestry-specific clinical LDL risk. The main finding of this study is that the LDL-related drug ranking was highly stable across ancestry-defined datasets, while the receptor-level explanation of the score was strongly sensitive to the weighting scheme. The weighted and uniform models produced mean cross-ancestry rank correlations of 0.996 and 0.995, respectively. The weighted top-10 set was identical across all ancestry pairs, whereas the uniform model produced a lower mean top-10 Jaccard value of 0.758. This pattern suggests that ancestry-specific LDL TWAS inputs caused relatively small perturbations to the broad drug ordering but more visible changes when the model was allowed to rely on equal receptor priors.

The gene-level results were even more consistent. All 39 genes represented in the exported direction-consistency analysis changed contribution share in the same direction across all five datasets. DRD2, DRD3, and DRD4 consistently gained relative share, while HRH1, HTR2A, and HTR2C consistently lost share. DRD2 was the leading uniform-model contributor in every ancestry. These findings support a stable direction of model sensitivity rather than an ancestry-specific effect driven by a single dataset.

The result is notable in the context of multi-ancestry lipid genetics, where ancestry differences in allele frequencies, linkage disequilibrium, prediction-model performance, and fine-mapping resolution can affect gene-level association patterns [14-16]. However, the interpretation must remain narrow. The five analyses shared the same drug list, Ki values, defined daily doses, receptor mappings, contribution formula, and prior dictionary. The perfect directional consistency therefore reflects both the ancestry-specific TWAS inputs and a common pharmacological structure. It demonstrates transancestral stability of model behavior, not transancestral validation of causal receptor mechanisms.

The appropriate conclusion is that ancestry-specific LDL TWAS inputs produced only modest changes in relative drug ranking within this framework. This supports evaluation of a common reference ranking for research communication, but it does not establish that ancestry-specific clinical adjustment is unnecessary. Clinical generalizability requires patient-level outcome validation in diverse populations.

Clinical context and prior dependence

The literature-weighted model had greater qualitative concordance with known metabolic hierarchies than the uniform model, but its agreement with clinical evidence was qualitative rather than exact. Clozapine remained the highest-ranked drug across all ancestry datasets, and olanzapine remained in the upper tier. These findings are compatible with clinical evidence showing greater metabolic liability for clozapine and olanzapine, including greater average adverse effects on weight, glucose, and selected lipid outcomes than for several newer agents [1,2,4]. The model also placed aripiprazole lower than many of the drugs with stronger histaminergic and serotonergic contributions.

The weighted model nevertheless ranked chlorpromazine second and asenapine third in the cross-ancestry consensus ordering, while ziprasidone occupied a mean rank of 5.80 in the broader weighted summary. These placements should not be presented as a validated clinical hierarchy. They show that the model retains a mixture of pharmacological affinity, defined daily dose, TWAS evidence, nominal significance bonuses, and normalization choices. External clinical studies provide context for the output but do not validate the exact order of all 52 drugs.

Removing the literature priors made this distinction clearer. The uniform model moved ziprasidone, aripiprazole, and haloperidol toward numerically smaller ranks, which correspond to higher predicted risk because rank 1 denotes the highest predicted risk. It also moved olanzapine and quetiapine toward numerically larger ranks. These movements are discordant with the usual clinical interpretation of these drugs and demonstrate that a uniform model should not be used alone for treatment selection or patient counselling.

The class-level reallocation helps explain the behavior. The weighted model placed substantial relative emphasis on histaminergic and serotonergic systems, especially HRH1, HTR2C, and HTR2A. The clinical relevance of H1 and 5-HT2C mechanisms is supported by receptor-binding and metabolic literature [9-12]. The network meta-analysis [2] also found that H1, M1, and M3 receptor occupancies were associated with greater glyco-metabolic deterioration. The weighted model therefore embeds prior knowledge that is broadly consistent with known antipsychotic metabolic patterns. This makes it more interpretable, but also partly circular.

Dual-reporting recommendation

The results support reporting both models rather than selecting one model and treating it as definitive. We recommend dual reporting: the literature-weighted model as the clinically informed reference and the uniform model as a sensitivity analysis of receptor-prior assumptions. The literature-weighted model should be used as the primary clinically informed reference because it incorporates established pharmacological knowledge and better preserves the expected prominence of agents with substantial metabolic liability. It may be useful for comparative research communication and for organizing hypotheses about drug-level metabolic burden.

The weighted model should not be described as clinically validated. Its receptor weights were manually specified from prior literature, its dose term is a defined daily dose proxy rather than a measure of individual exposure, and its output has not been calibrated against longitudinal LDL, weight, glucose, diabetes, metabolic syndrome, or cardiovascular outcomes. Clinical guidance supports monitoring and management of metabolic risk during antipsychotic treatment, but it does not establish this score as a clinical decision instrument [6-8].

The uniform model should be retained as a sensitivity and discovery-oriented analysis. It asks how much the drug ranking and receptor attribution depend on the literature priors. It can reveal genes and pathways that are suppressed by prior weighting, including DRD2, DRD3, DRD4, HTR2B, and ADRB1. It should not be described as unbiased, because equal weighting is itself a prior assumption. It also restores contribution from genes absent from the weighted dictionary, so the comparison is a change in both the strength and the scope of the receptor prior.

A dual-reporting framework is therefore more informative than a single model. The weighted model communicates the consequences of incorporating established pharmacological evidence. The uniform model reveals how the conclusions change when that evidence is intentionally withheld. Agreement between the models supports robustness. Disagreement identifies areas where mechanistic interpretation is prior-dependent.

HRH1, DRD2, and ADRB1 as hypotheses

HRH1 was the strongest ranking-sensitive component in the leave-one-gene-out analysis. This result is compatible with prior evidence linking H1 receptor affinity to weight gain and with clinical pharmacology studies associating H1 occupancy with metabolic deterioration [2,9]. The present analysis extends that observation by showing how removing HRH1 changes the relative positions of particular drugs within the reconstructed LDL ranking. It does not show that HRH1 is the sole or dominant causal mediator of LDL change.

DRD2 became the leading gene-level reconstructed contribution under uniform weighting in every ancestry. This finding is biologically plausible because dopaminergic pathways influence reward, feeding behavior, endocrine signaling, and metabolic regulation. Nevertheless, the result is generated by the interaction of Ki values, defined daily dose, TWAS evidence, p-value scaling, and the removal of differential priors. It should therefore be treated as a testable hypothesis rather than evidence that D2-related mechanisms are clinically more important than histaminergic or serotonergic mechanisms.

ADRB1 was the strongest high-TWAS/low-weight exploratory, single-ancestry candidate, with an absolute z-score of 6.079 in the European dataset and a literature weight of 0.08. Adrenergic signaling has established roles in adipose-tissue metabolism and energy expenditure, and beta-1 adrenergic receptors may contribute to human brown-adipocyte metabolic activity [24,25]. These studies provide biological plausibility but do not demonstrate that ADRB1 mediates antipsychotic-associated LDL changes. The present ADRB1 signal was supported in only one ancestry, so it does not yet meet a multi-ancestry replication standard. Independent TWAS replication, colocalization, tissue-specific analysis, fine-mapping, and functional testing are needed before assigning clinical or causal importance.

Relation to the broader series

The LDL analysis complements the earlier comparison of four TWAS transformations and the receptor-prior reallocation analysis. The scaling-method study showed that the choice of TWAS transformation mainly changed score magnitude and had a smaller effect on drug ordering [19]. The prior-removal analysis showed that rank stability can coexist with substantial changes in receptor-level explanation and identified ADRB1 as an underweighted candidate [20]. The HDL Stage 3 analysis examined whether these effects were stable across ancestry-defined datasets and found a similar distinction between stable model behavior and clinically discordant individual rankings [21].

The LDL results strengthen the generalizability of that conceptual framework across lipid traits. The recurring pattern is not that every receptor finding is identical between LDL and HDL, but that removing priors consistently exposes a difference between ranking robustness and mechanistic robustness. That distinction is relevant to other pharmacological models that combine empirical drug features with literature-derived biological weights.

Limitations

Several limitations should be considered. Most importantly, this score is a computational prioritization index rather than a validated clinical risk predictor. No patient-level LDL changes, triglyceride changes, weight trajectories, glucose outcomes, incident diabetes, metabolic syndrome diagnoses, or cardiovascular events were used for validation.

Second, defined daily dose is a standardized dose proxy. It does not represent the prescribed dose for an individual patient, treatment adherence, treatment duration, plasma exposure, receptor occupancy, or tissue concentration. The Ki-DDD term is therefore useful for comparative modeling but cannot be interpreted as a direct estimate of pharmacological exposure.

Third, the pipeline used minimum Ki aggregation. Selecting the lowest reported Ki value may be useful for identifying potential binding interactions, but it can overemphasize an isolated measurement and may not represent the central affinity of a drug across assays. A median, mean, or geometric-mean sensitivity analysis was not included in the saved Stage 3 run. Alternative aggregation strategies, including median, mean, or hierarchical models, should be evaluated.

The internal nature of the Ki database limits record-level reproducibility. In addition, the literature-derived receptor-weight dictionary is not independent of the pharmacological evidence used to interpret metabolic liability; its use therefore introduces a form of circularity into the weighted model. Conversely, the uniform model is not assumption-free because equal weighting is itself a prior.

Fourth, fuzzy ligand matching and generic receptor labels introduce uncertainty. A generic alpha-1 label was mapped to ADRA1A, a generic alpha-2 label to ADRA2A, and a generic muscarinic label to CHRM1. These operational choices may not accurately represent subtype-specific binding. The final workflow should preserve alternative matches and manual adjudication status.

Fifth, missing TWAS values were imputed. The upstream runs contained 97 to 175 nominally significant rows and approximately 210 to 239 imputed TWAS rows per ancestry. The reconstruction also reapplied mean negative-log10 p-value imputation for missing scored genes. This procedure avoids automatically penalizing drugs with incomplete coverage, but it may reduce genuine heterogeneity and introduce common contribution into rows without directly observed TWAS values. The saved Stage 3 outputs did not include a separate analysis excluding imputed rows, so the magnitude of this influence could not be quantified.

Sixth, the TWAS transformations used p-values and absolute z-scores. They therefore ignored the direction of association. The score identifies genes with strong statistical evidence but cannot determine whether genetically predicted expression is associated with higher LDL, lower LDL, or a clinically adverse direction. The term “LDL-associated contributor” is more appropriate than “LDL risk gene.”

Seventh, the nominal p-value threshold of .05 was not corrected for multiple testing. The high-TWAS/low-weight screen was exploratory and was not equivalent to genome-wide significance, false-discovery-rate control, colocalization, or causal evidence.

Eighth, the five ancestry analyses were not fully independent replications of the complete model because they shared the same Ki data, defined daily dose values, receptor mappings, drug list, and prior dictionary. The strong consistency of the rank changes therefore provides evidence of structural reproducibility but cannot by itself establish that the same causal mechanisms operate in every ancestry.

Ninth, the leave-one-gene-out calculation used reconstructed receptor contributions and did not fully reproduce all drug-level multipliers in the saved final score. It should therefore be described as sensitivity of the reconstructed contribution-based ranking. Future analyses should recompute the complete final score after gene removal.

The saved Stage 3 outputs did not preserve complete package-version metadata, which limits exact reproduction of the computational environment. The negative-log10 p-value cap of 25 was used as a numerical safeguard, but its sensitivity was not formally tested in the saved Stage 3 run.

Finally, the present Stage 3 output did not contain an independent patient-level clinical comparator for LDL. Clinical alignment was therefore qualitative. A future study should prespecify a comparator based on independent clinical data and evaluate discrimination, calibration, rank agreement, and outcome-specific performance.

## Conclusions

This Stage 3 analysis evaluated a computational prioritization index, not a validated patient-level clinical risk predictor, by examining the sensitivity of an integrated Ki-DDD-TWAS antipsychotic metabolic-risk framework to literature-derived receptor priors in ancestry-defined LDL datasets. The main finding was a clear separation between the stability of drug ordering and the stability of mechanistic attribution. Drug ordering remained highly stable across ancestry-defined datasets in both models, although uniform weighting reduced the stability of membership in the highest-ranked set. Clozapine remained the highest-ranked drug across all ancestry-defined datasets, and olanzapine remained in the upper tier of the weighted model. At the receptor level, removing the literature priors produced a substantial and directionally consistent redistribution of contribution away from histaminergic and serotonergic systems and toward dopaminergic receptors. DRD2 remained the leading contributor under uniform weighting across all ancestry-defined datasets. HRH1 was the most ranking-sensitive gene in the weighted leave-one-gene-out analysis.

These findings support the interpretation that receptor priors stabilize the clinical interpretability of the model while also shaping its mechanistic conclusions. The uniform model is valuable for sensitivity analysis and hypothesis generation, but its clinically discordant movements should prevent its use as a standalone treatment-ranking tool. ADRB1 is a plausible candidate for further research because of its strong LDL TWAS signal and low literature weight, but it lacked support across the ancestry datasets and should not be treated as a confirmed causal mechanism. Because the analyses shared pharmacological inputs across ancestries and lacked patient-level clinical validation, the findings describe model behavior rather than clinical risk. We recommend dual reporting: the literature-weighted model as the clinically informed reference and the uniform model as a sensitivity analysis of receptor-prior assumptions. The findings demonstrate sensitivity of mechanistic attribution to receptor-prior assumptions while drug ordering remains relatively stable; they do not establish clinical risk, causality, or treatment-ranking validity.

## Appendices

Appendix 1

Data and Code Availability

The WHO Anatomical Therapeutic Chemical (ATC) classification system/DDD input is publicly accessible through the source cited in reference 22. The analysis code is provided as supplementary material in the file 080JvG(rank_changes_without_w)_TWAS_LDL_Risk_x_Antipsychotics_Ki.ipynb and is also publicly available in the GitHub repository “080JvG-Clinical-Rank-Shifts-and-Transancestral-Stability-in-Antipsychotic-LDL-Risk-Modelling” [26]. The supplementary code contains the N05A drug-list extraction, ligand matching, Ki-DDD score construction, TWAS integration, weighted and uniform model analyses, downstream reconstruction, and Stage 3 transancestral analyses.

The file KiDatabase_2026-06-22.csv, the receptor-weight dictionary, and the receptor-to-gene map were internal analysis files and were not deposited as a complete public dataset. The main reconstruction rules are reported here: lower-case, whitespace-normalized ligand names, RapidFuzz WRatio matching at a threshold of 80, parsing of Ki values in nanomolar units, minimum-Ki aggregation, deterministic receptor-to-gene mapping, the specified receptor-weight dictionaries, mean negative-log10 p-value imputation, and the contribution formula described below. If the full Ki file cannot be released, record-level ligand, receptor, and Ki information would be required for independent reconstruction.
